# Time to death from cervical cancer and its predictors in hospitalized patients: a survival approach study in Mato Grosso, Brazil

**DOI:** 10.1186/s12957-024-03518-y

**Published:** 2024-10-09

**Authors:** Sancho Pedro Xavier, Kátia Moreira da Silva, Noemi Dreyer Galvão, Marco Aurélio Bertúlio das Neves, Adila de Queiroz Neves Almeida, Ageo Mario Cândido da Silva

**Affiliations:** 1 Institute of Collective Health, Federal University of Mato Grosso, Av. Fernando Correa da Costa, nº 2367 - Bairro Boa Esperança, Cuiabá, Mato Grosso 78060-900 Brazil; 2 State Secretary of Health of Mato Grosso, Cuiabá, Mato Grosso Brazil

**Keywords:** Cervical cancer, Survival, Prognostic factors, Hospitalized patients, Mato Grosso

## Abstract

**Background:**

Cervical cancer (CC) is a serious public health concern, being the fourth most common cancer among women and a leading cause of cancer mortality. In Brazil, many women are diagnosed late, and in Mato Grosso, with its geographical diversity, there are specific challenges. This study analyzed hospital survival and its predictors using data from the Hospital Information System (SIH) of the Unified Health System (SUS) in Mato Grosso from 2011 to 2023.

**Methods:**

Cox regression and Kaplan-Meier models were applied to determine survival time and identify mortality predictors. The adjusted Hazard Ratio (AHR) with a 95% Confidence Interval (CI) was used to measure the association between the factors analyzed.

**Results:**

The hospital mortality rate was 9.88%. The median duration of hospitalization was 33 days (interquartile range [IQR]: 12–36), with a median survival of 43.7%. Patients were followed up for up to 70 days. In the multivariable Cox model, after adjusting for potential confounders, the risk of death during hospitalization was higher in patients aged 40–59 years (AHR = 1.39, *p* = 0.027) and 60–74 years (AHR = 1.54, *p* = 0.007), in the absence of surgical procedures (AHR = 4.48, *p* < 0.001), in patients with medium service complexity (AHR = 2.40, *p* = 0.037), and in the use of ICU (AHR = 4.97, *p* < 0.001). On the other hand, patients with hospital expenses above the median (152.971 USD) showed a reduced risk of death (AHR = 0.21, *p* < 0.001).

**Conclusion:**

This study highlights that hospitalized CC patients have reduced survival, underscoring the need for interventions to improve care, including strategies for early diagnosis and expanded access to adequately resourced health services.

**Supplementary Information:**

The online version contains supplementary material available at 10.1186/s12957-024-03518-y.

## Introduction

Cervical cancer (CC) is the fourth most common type of cancer among women worldwide [1] and one of the leading causes of cancer-related deaths, representing a significant impact on patients, families, and societies [2]. Globally, it is estimated that more than one million women have the disease, many of whom are undiagnosed or lack access to treatment that could cure them or improve their survival [2]. Therapeutic options can be systemic (chemotherapy and hormone therapy) and locoregional (surgery or radiotherapy), ranging from a single surgery to a combination of radiotherapy and chemotherapy, depending on the clinical staging of the disease at diagnosis, as well as individual factors such as age [3, 4].

CC is responsible for approximately 7.5% of all cancer deaths among women, with the highest incidence occurring in the age group of 35 to 65 years [5]. The magnitude and distribution of CC vary significantly among different countries worldwide [6]. In Brazil, it is a significant concern, as women are often diagnosed with the disease at an advanced stage [7]. In 2020, approximately 16,710 new cases of CC were recorded in the country, with an incidence rate of 16.35 per 100,000 women and a mortality rate of 5.33 per 100,000 women [8]. In the state of Mato Grosso, CC was the second most common neoplasm among women in 2020, with 12.43 cases per 100,000 women, and an estimated 12.33 cases for 2023 [8]. Early detection of the disease has a significant impact on mortality and survival rates. Women diagnosed with CC at stage I have a five-year survival rate of about 90%, while survival rates at stages II and III are 50% and 10%, respectively [9, 10]. A study conducted in the city of Cuiabá, in the state of Mato Grosso, Brazil, identified a five-year specific survival rate of 90.0%, based on data from the Population-Based Cancer Registry [11]. Advances in screening, genital hygiene, vaccination, and treatment have played a crucial role in reducing cancer mortality [12, 13]. Studies show that patients who have never undergone screening have a significantly worse survival rate compared to those who have been screened [13, 14].

Survival analysis is a term used to describe data that measures the time until the occurrence of a particular event of interest. These data evaluate the duration of survival of a group of patients after diagnosis or treatment [15]. Survival care is crucial for providing high-quality care to patients at all stages of cancer, in addition to early diagnosis and standard treatments. In the present study, the event of interest is the survival time of hospitalized CC patients from the day they were admitted for hospitalization. In Mato Grosso, as far as is known, the number of studies focusing on the survival of CC patients is incipient, and no research was found that analyzed the survival of patients during hospitalization and their prognoses. This study aimed to analyze the survival of these patients after admission between 2011 and 2023 for hospitalization and to identify their predictors.

## Methods

### Study design and data collection

A retrospective cohort study was conducted using secondary data obtained from the Hospital Information System (SIH) of the Data Repository of Information Systems of the State Health Department of Mato Grosso (DwWeb SES-MT) of the Unified Health System (SUS). Established in 1991, the SIH is an administrative database that records hospital admissions in the SUS through the Hospital Admission Authorization (AIH) form, primarily for reimbursing hospital expenses and providing data for morbidity and mortality studies [16]. The data is open access and freely available online at: http://appweb3.saude.mt.gov.br/dw/pesquisa/tema. In accordance with ethical guidelines [17], formal ethical approval was not required. All procedures adhered to the established ethical principles for scientific research and respected the privacy and confidentiality of the data.

### Population and sample size

The study population included all patients diagnosed with CC (topographic code C53) who were admitted for hospitalization between 2011 and 2023. All observations (cases) with missing data were excluded from the analysis. In total, 3,493 hospitalized patients were included in the analysis.

### Variables

The outcome variable was the time to death of CC patients, measured in days from the time of hospital admission. Patient death was coded as an event (1), while patients who did not die during hospitalization were censored (0). The predictor variables analyzed included: patient age groups (16–39, 40–59, and 60+); skin color; municipality of residence; cancer topographic type, categorized as malignant neoplasm of the cervix uteri, not otherwise specified (NOS) (code C53) / Cervix uteri, unspecified (C53.9), Ectocervix (C53.1), Endocervix (C53.0) and Invasive lesion (C53.8). The NOS category was combined with the unspecified category based on the assumption that both NOS and unspecified indicate a lack of specific detailing of the tumor location. Medical procedures performed during hospitalization were categorized as clinical or surgical; hospitalization period (Covid-19 pandemic and non-pandemic periods); hospitalization nature (emergency or elective); complexity of services provided (medium and high); ICU admission; and total hospital expenses, categorized in relation to the median value (up to the median and above the median), with conversion from Brazilian reais to US dollars, as shown in Table 1. The SUS is composed of basic units and services of medium and high complexity [18]. Medium complexity care provides services with specialized professionals and technology for diagnosis and treatment. High complexity care, on the other hand, involves procedures requiring advanced technology and high costs, offering specialized services that are integrated with other levels of health care, such as primary and medium complexity care [19].

### Statistical analysis

For qualitative variables, descriptive analyses were used, expressing absolute and relative frequencies. For quantitative variables, the Shapiro-Wilk test was applied to check the normality of the data, with results presented in medians and interquartile ranges (IQR) of 25% and 75%. Survival models were used to analyze the time until the occurrence of the event (in-hospital death), using non-parametric approaches to handle censored data. Survival estimates were calculated using the Kaplan-Meier method, and survival differences between groups were assessed using the Log-rank test [20]. Additionally, the Tarone-Ware and Peto-Prentice tests were applied to examine differences between survival curves [21]. Survival data were modeled considering two main functions: (i) the survival function $\:S\left(t\right)$, which represents the probability that patients survive beyond a time $\:t$ after hospital admission, and the hazard function $\:h\left(t\right)$ [15], which indicates the instantaneous rate of event occurrence (death) for individuals who have already survived up to time $\:t$ [15, 22].

Cox proportional hazards regression models, both univariable (CHR) and multivariable (AHR), were applied to assess the impact of predictor variables on patient survival times. Variables that showed statistically significant p-values in the Log-rank, Tarone-Ware, or Peto-Prentice tests were included in the model. Hazard ratios (HR) and 95% confidence intervals (CI) were calculated. In the final model, all variables with at least one category with a p-value < 0.25 were included [23, 24]. To select the best-fitting model, we used the Akaike Information Criterion (AIC), which balances model fit with complexity by penalizing models with more parameters. Lower AIC values indicate a better balance between model fit and complexity [25]. To verify the proportional hazards assumption, the Goodness-of-Fit (GOF) test was used through the Schoenfeld residual test (see supplements: **S3**). A p-value < 0.05 was considered statistically significant. Data analysis was performed using R software version 4.4.0 (https://www.r-project.org/) and RStudio version 2024.04.2 + 764 (released on June 10, 2024) (https://posit.co/download/rstudio-desktop/).

## Results

### Sociodemographic and clinic characteristics of the study participants

According to Table 1, of the 3,493 patients hospitalized with CC, the majority (46.7%) were in the age group 40–59 years, followed by 16–39 years (32.8%) and 60 or older (20.5%). The median age was 45 years, with an interquartile range of 37.3 to 57 years. Although the majority of patients (51.0%) resided in municipalities in the interior of the state of Mato Grosso, a significant portion lived in the municipalities of Cuiabá (23.6%) and Várzea Grande (10.1%).

The most common topographies were malignant neoplasm NOS/unspecified (41.6%), invasive lesion (23.5%), endocervix (25.3%), and ectocervix (9.6%). Regarding medical procedures performed, 62.5% of patients underwent surgical procedures, and 37.5% underwent non-surgical procedures. The hospitalization period was also analyzed, with 85.0% of hospitalizations occurring during the COVID-19 pandemic period and 15.0% in non-pandemic.

On the nature of hospitalization, most hospitalizations (51.3%) were elective. In terms of the complexity of services provided, 29.0% of the patients were treated in high-complexity units, and 71.0% in medium-complexity units. Concerning the need for intensive care, 91.5% of patients did not require ICU, while 8.5% did. Regarding the total cost of hospitalization, patients were equally divided, with 49.3% of cases having a total value up to the median (152.971 USD) and 50.7% above the median.

### Time to death by Kaplan-Meier estimates of the failure function

**Fig. 1:**
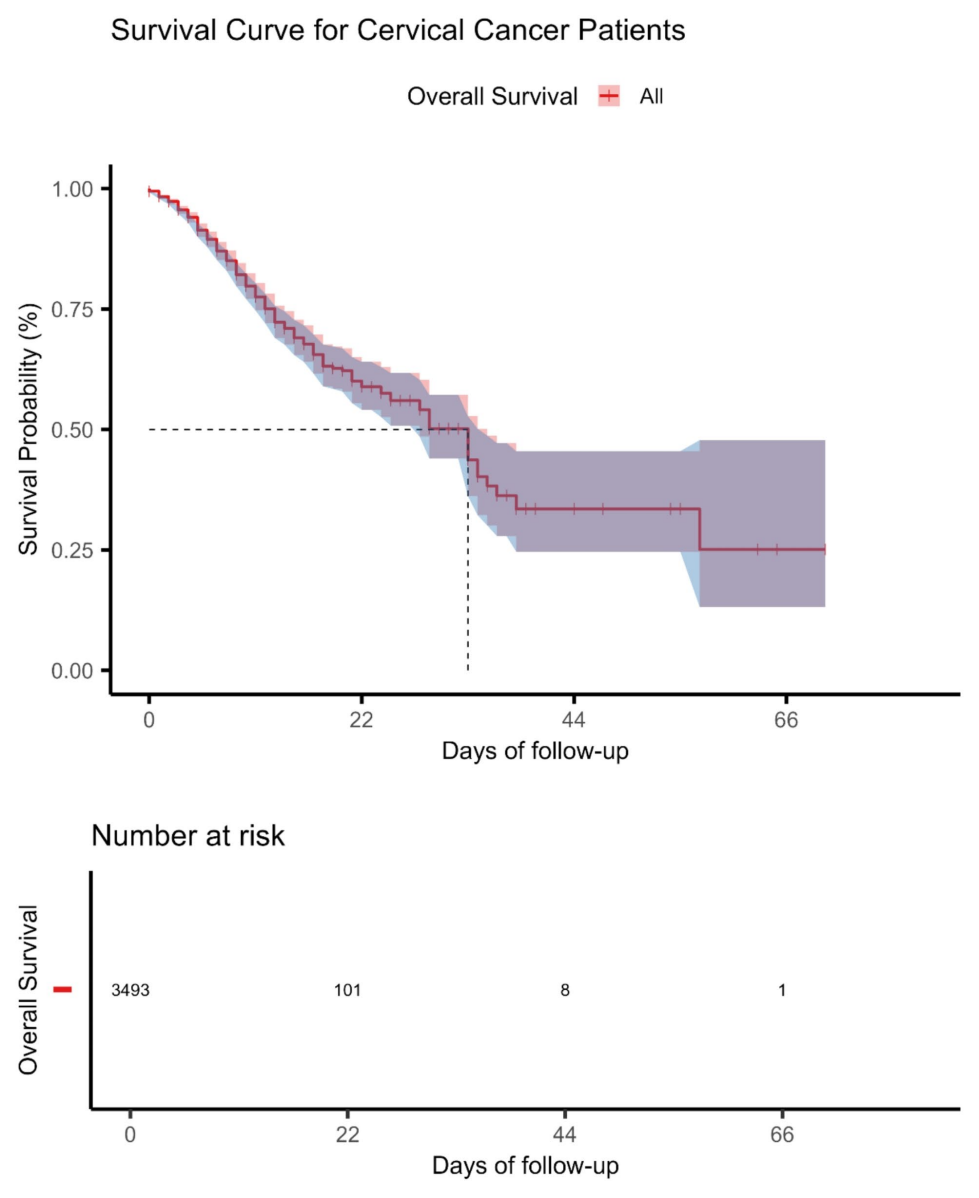
Survival curve for cervical cancer patients

The median hospitalization time until death was 33 days (95% CI: 28, 35), with an interquartile range of 12 to 36 days. The minimum hospitalization time was 0 days and the maximum 70 days. The hospital mortality rate for CC was 9.88%. The overall and median survival rates were 25.1% (95% CI: 13.2, 47.8) at 70 days and 43.7% (95% CI: 36.2, 52.8) at 33 days, respectively, as illustrated in Fig. 1 and Table S1.

### Analysis of factors associated with patient survival

The analysis of factors associated with the survival of CC patients, as described in Table 1, shows significant differences between the survivor and censored groups. Patients in the age group 16–39 years had the highest survival rate (94.4%), while the rate progressively decreased with increasing age, being lowest for those aged 60 years or older (83.9%). These differences were statistically significant (*p* < 0.001).

Survival was slightly higher among white patients (91.3%) compared to non-whites (89.9%), but these differences were not statistically significant (*p* > 0.05). The survival rate was highest for patients with endocervical cancer (97.3%) and lowest for those with malignant neoplasm NOS/unspecified (86.1%), with statistically significant differences (*p* < 0.001). Patients who underwent surgical medical procedures had a significantly higher survival rate (94.9%) compared to those who did not undergo surgery (88.7%) (*p* < 0.001). Survival rates were slightly higher during the non-pandemic period (90.4%) compared to the pandemic period (88.4%), but these differences were not statistically significant (*p* > 0.3).

Patients admitted on an emergency basis had a significantly higher survival rate (95.5%) compared to elective admissions (85.0%) (*p* < 0.001). Patients in high complexity services had a much higher survival rate (99.3%) compared to those in medium complexity (86.4%) (*p* < 0.001).

Survival was significantly lower for patients who required ICU care (72.0%) compared to those who did not require ICU care (91.8%) (*p* < 0.001). Patients whose total hospitalization cost was above the median had a higher survival rate (92.0%) compared to those whose cost was below the median (88.3%) (*p* < 0.001).

**Table 1:** Analysis of Factors Associated with Survival from Cervical Cancer in Hospitalized patients

Predictors		Survival status		*p* - value		
	Total *n* = 3493	Alive *n* = 3148	Censored *n* = 345	Log-rang	Tarone-Ware	Gehan-Breslow
Age (in Years)						
16–39	1147 (32.8)	1083 (94.4)	64 (5.6)			
40–59	1630 (46.7)	1467 (90.0)	163 (10.0)	*p* < 0.001	*p* < 0.001	*p* < 0.001
60 or more	716 (20.5)	598 (83.5)	118 (16.5)			
Skin Color						
White	634 (18.2)	579 (91.3)	55 (8.7)			
Non-White	2859 (81.8)	2569 (89.9)	290 (10.1)	*p* = 1.00	*p* = 0.906	*p* = 0.762
Municipality of Residence						
Cuiabá	825 (23.6)	734 (89.0)	91 (11.0)			
Rondonópolis	312 (8.9)	265 (84.9)	47 (15.1)			
Sinop	221 (6.3)	209 (94.6)	12 (5.4)	*p* = 0.100	*p* = 0.0363	*p* = 0.052
Várzea grande	353 (10.1)	303 (85.8)	50 (14.2)			
Other	1782 (51.0)	1637 (91.9)	145 (14.2)			
Type of Cancer by Topography						
NOS/unspecified	1452 (41.6)	1250 (86.1)	202 (13.9)			
Ectocervix	335 (9.6)	331 (98.8)	115 (14.0)			
Endocervix	885 (25.3)	861 (97.3)	4 (1.2)	*p* < 0.001	*p* < 0.001	*p* < 0.001
Invasive lesion	821 (23.5)	706 (86.0)	24 (2.7)			
Medical Procedure						
Surgical	2183 (62.5)	2149 (94.9)	34 (5.1)			
Non-surgical	1310 (37.5)	999 (88.7)	311 (11.3)	*p* < 0.001	*p* < 0.001	*p* < 0.001
Hospitalization Period						
Non-pandemic	2969 (85.0)	2685 (90.4)	284 (9.6)			
Pandemic	524 (15.0)	463 (88.4)	61 (11.6)	*p* = 0.300	*p* = 0.140	*p* = 0.164
Hospitalization Nature						
Urgent/Emergency	1700 (48.7)	1624 (95.5)	76 (4.5)			
Elective	1793 (51.3)	1524 (85.0)	269 (15.0)	*P* < 0.001	*P* < 0.001	*P* < 0.001
Service Complexity						
High	1013 (29.0)	1006 (99.3)	7 (0.7)			
Medium	2480 (71.0)	2142 (86.4)	338 (13.6)	*p* < 0.001	*p* < 0.001	*p* < 0.001
ICU admission						
No	3197 (91.5)	2935 (91.8)	262 (8.2)	*p* = 0.008	*p* < 0.001	*p* < 0.001
Yes	296 (8.5)	213 (72.0)	83 (28.0)			
Total Hospital Cost (median* = 152,971 USD)						
Up to median	1722 (49.3)	1517 (88.1)	205 (11.9)	*p* < 0.001	*p* < 0.001	*p* < 0.001
Above median	1771 (50.7)	1631 (92.1)	140 (87.9)			

* Total hospital cost was converted to US dollars

### Parametric survival model distribution

**Fig. 2:**
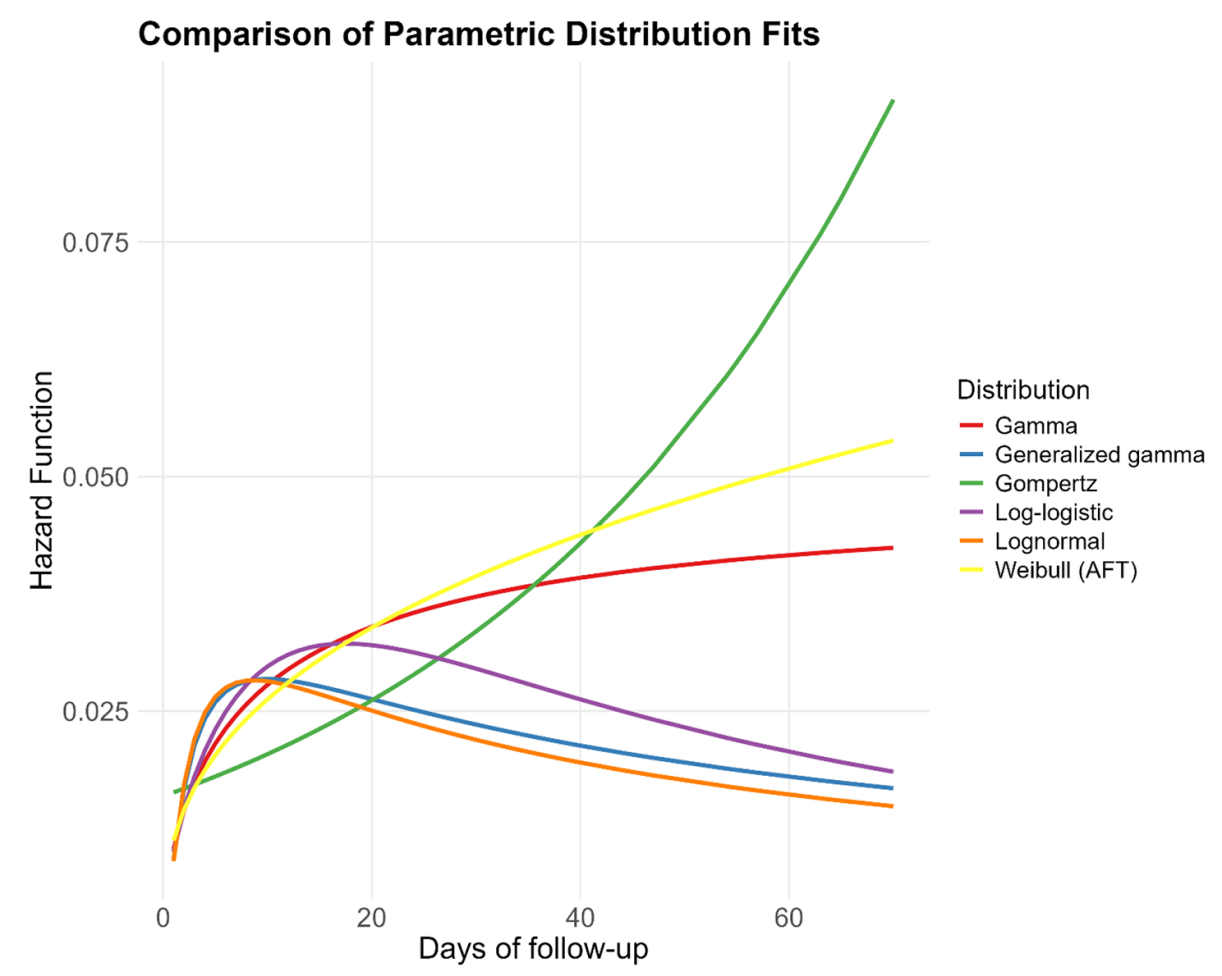
Comparison of parametric distribution fits

The AIC results suggest a trend towards a log-normal distribution for the data, after excluding zero hospital stay times, as shown in Fig. 2; Table 2. However, to include zero hospital stay times in the analyses, a semiparametric distribution was applied.

**Table 2:** AIC values of the parametric Cox models

Distribution name	AIC
Lognormal	3139.
Generalized gamma	3141.
Log-logistic	3148.
Gamma	3156.
Weibull (AFT)	3165.
Gompertz	3217.

### Predictors of mortality in patients with cervical cancer

The univariable analysis of mortality predictors in patients with CC, using Cox regression, included variables such as age, medical procedures performed, healthcare service complexity, ICU admission, and hospital costs, as shown in Table 3.

In the reduced multivariable analysis, adjusted for potential confounding factors, age remained a significant predictor of mortality. Patients aged 40–59 years (AHR = 1.39, 95% CI: 1.04, 1.86, *p* = 0.027) and 60–74 years (AHR = 1.54, 95% CI: 1.13, 2.11, *p* = 0.007) had an increased risk compared to the 16–39 age group, representing an approximately 39% and 54% increase in risk, respectively. Patients who did not undergo surgical procedures had a significantly higher mortality risk (AHR = 4.48, 95% CI: 3.00, 6.68, *p* < 0.001) compared to those who underwent surgery, representing an approximately 348% increase in risk.

The medium complexity of services provided continued to show an elevated risk (AHR = 2.40, 95% CI: 1.07, 5.40, *p* = 0.037) compared to high complexity, and ICU admission was a strong predictor of mortality (AHR = 4.97, 95% CI: 3.55, 6.97, *p* < 0.001). Hospital costs above the median were associated with a significantly reduced risk of mortality (AHR = 0.21, 95% CI: 0.16, 0.29, *p* < 0.001).

**Table 3:** Hazard ratio (HR) estimates for mortality in patients with cervical cancer using cox regression

Predictors	Univariable model		Multivariable model		Multivariable model reduced	
	CHR (IC 95%)	*p* – value	AHR (IC 95%)	*p* – value	AHR (IC 95%)	*p* – value
Age (in Years)						
16–39	Ref		Ref		Ref	
40–59	1.56 (1.17, 2.08)	*p* = 0.003	1.39 (1.04, 1.87)	*p* = 0.026	1.39 (1.04, 1.86)	*p* = 0.027
60 or more	2.22 (1.64, 3.02)	*p* < 0.001	1.55 (1.13, 2.12)	*p* = 0.006	1.54 (1.13, 2.11)	*p* = 0.007
Municipality of Residence						
Other	Ref		Ref		Ref	
Cuiabá	1.06 (0.81, 1.38)	*p* = 0.671	1.21 (0.92, 1.57)	*p* = 0.167	1.21 (0.93, 1.58)	*p* = 0.157
Sinop	1.04 (0.57, 1.87)	*p* = 0.901	1.34 (0.74, 2.45)	*p* = 0.333	1.34 (0.74, 2.43)	*p* = 0.336
Várzea grande	1.12 (0.81,1.55)	*p* = 0.488	1.22 (0.88, 1.69)	*p* = 0.241	1.22 (0.88, 1.69)	*p* = 0.228
Rondonópolis	1.54 (1.10, 1.55)	*p* = 0.011	1.42 (0.99, 2.02)	*p* = 0.052	1.41 (0.99, 2.00)	*p* = 0.053
Type of Cancer by Topography						
Ectocervix	Ref		Ref		Ref	
NOS/unspecified	4.19 (1.55, 11.30)	*p* = 0.005	2.00 (0.74, 5.45)	*p* = 0.173	2.00 (0.74, 5.43)	*p* = 0.174
Endocervix	1.62 (0.56, 4.68)	*p* = 0.369	1.39 (0.48, 4.03)	*p* = 0.534	1.39 (0.48, 4.03)	*p* = 0.541
Invasive lesion	3.95 (1.45, 10.73)	*p* = 0.007	1.79 (0.65, 4.03)	*p* = 0.258	1.79 (0.65, 4.92)	*p* = 0.258
Medical Procedure						
Surgical	Ref		Ref		Ref	
Non-surgical	6.99 (4.88, 10.03)	*p* < 0.001	4.59 (2.97, 6.78)	*p* < 0.001	4.48 (3.00, 6.68)	*p* < 0.001
Hospitalization Period						
Non-pandemic	Ref					
Pandemic	1.17 (0.88, 1.54)	*p* = 0.277	--------------------	----------	--------------------	----------
Hospitalization Nature						
Urgent/Emergency	Ref		Ref			
Elective	2.13 (1.65, 2.76)	*p* < 0.001	0.97 (0.74, 1.27)	*p* = 0.816	--------------------	----------
Service Complexity						
High	Ref		Ref		Ref	
Medium	10.84 (5.12, 22.97)	*p* < 0.001	2.43 (1.08, 5.46)	*p* = 0.032	2.40 (1.07, 5.40)	*p* = 0.037
ICU admission						
No	Ref		Ref		Ref	
Yes	1.41 (1.09, 1.81)	*p* = 0.008	4.98 (3.55, 6.99)	*p* < 0.001	4.97 (3.55, 6.97)	*p* < 0.001
Total Hospital Cost						
Up to median	Ref		Ref		Ref	
Above median	0.25 (0.20, 0.32)	*p* < 0.001	0.21 (0.16, 0.29)	*p* < 0.001	0.21 (0.16, 0.29)	*p* < 0.001
AIC: chisq (p-value)			4275.82 **(*p* < 0.001)**		4273.45 **(*p* < 0.001)**	
Global test (phz): chisq (p-value)			37.16 **(*p* < 0.001)**		34.87 **(*p* < 0.001)**	

*Note: phz: proportional hazard assumption; The dashes or hyphens (“-----“) indicate that the variables were not included in the models (p-value ≥ 0.250); Ref: reference

## Discussion

The main objective of this study was to analyze the factors influencing the survival time of patients with CC during hospitalization, treated by the SUS in a state belonging to the Legal Amazon. The observed hospital mortality rate was 9.88%, with a median survival of 43.7% at 33 days of hospitalization. Several factors were identified after adjusting the model for possible confounders that influence the survival time of these patients during hospitalization. These factors include age, medical procedures performed, complexity of services, ICU admission, and hospital costs.

Patients hospitalized in the age groups of 40–59 and ≥ 60 years showed an increased risk of death of approximately 39% and 54%, respectively, compared to younger patients (≤ 39 years). This finding was consistent with previously published studies [1, 2, 26–30]. One possible explanation for this increased risk is the higher prevalence of chronic-degenerative diseases among patients in older age groups. Additionally, the clinical management of these patients may significantly contribute to this difference, as individuals in these age groups often receive less aggressive treatments and may be undertreated compared to younger patients [31]. This could occur due to concerns about these patients’ tolerance to intensive treatments and their side effects. Additionally, the tendency for diagnosis at more advanced stages of the disease in these age groups is a plausible hypothesis to explain the poorer prognosis observed. Older patients are often diagnosed at more advanced stages of the disease, possibly due to a fragmented health system, insufficient screening coverage, and early detection [31], which can delay diagnosis and worsen prognosis [32, 33]. On the other hand, some studies have presented inconsistent results, not identifying an association between age and survival [34, 35]. These discrepancies may be attributed to the variability in the methods applied during the studies, protocols adopted for patient care, healthcare system models, access to health services, as well as the social determinants of health of the population studied.

Non-surgical procedures were associated with an increased risk of death. These results were consistent with the findings of a study conducted in Bhutan, where non-surgical treatment types were independently associated with lower survival compared to patients undergoing surgery alone [36]. In the early stages, the recommended treatment is surgery [37], with or without radiotherapy, which may explain this finding, as the combination of radiotherapy and/or chemotherapy with surgery is recommended for more advanced stages of CC [38]. The Brazilian guidelines for addressing CC focus on screening and early diagnosis of precursor lesions, with the aim of providing outpatient treatment as a strategy to facilitate access to treatment for these lesions and prevent CC [39]. However, the waiting time between CC diagnosis and access to treatment in authorized oncology care facilities may be a determining factor for disease progression [40], influencing the choice of non-surgical treatment and resulting in a poorer prognosis for cure. Despite these established guidelines, clinical practice often involves a variety of therapeutic approaches. Non-surgical treatments are chosen in cases of risk factors associated with local recurrences, lymph node, parametrial, or surgical margin involvement, and when the cancer is locally advanced or in large tumors [36, 41]. When a patient has metastatic disease, therapeutic options are limited to chemotherapy or palliative treatment [36]. A randomized study comparing the survival of patients undergoing surgery versus radiotherapy did not identify significant differences [42], suggesting that there is no clear preferential treatment in terms of local disease control. This indicates that optimal treatment strategies depend on the benefits and disadvantages of each approach, as well as prognostic factors [42], including age, tumor size, and disease stage.

In this study, patients admitted to the ICU had an increased risk of death compared to patients not admitted. Research on the survival status of patients admitted to intensive care compared to those not admitted found that ICU-admitted patients had a significantly reduced survival rate [43], which aligns with our study’s results. Generally, ICU admission is considered appropriate for patients with malignancies due to specific indications such as postoperative care, complications caused by the malignant disease and/or its respective treatments, and non-cancer-related diseases or their respective therapies [44, 45]. Several risk factors can explain the reduced survival of cancer patients admitted to the ICU, such as the number of compromised organs [46], the need for mechanical ventilatory support [47], the consequent risk of ventilator-associated pneumonia [48], and the presence of sepsis, due to the compromised capacity of the immune system to fight infections [49–51]. Additionally, the survival of cancer patients admitted to the ICU can also be influenced by the treatment phase they are in, as patients in advanced stages of treatment, or those with recurrence or metastasis, tend to have a poorer prognosis [49–51]. This suggests that it is crucial to consider these factors when deciding on ICU admission for oncology patients.

Our results showed an association between hospital costs above the median and a significantly reduced risk of mortality for hospitalized patients with CC. One possible explanation for this association is that higher hospital costs often reflect access to advanced and comprehensive treatments, better hospital conditions, and more rigorous medical follow-ups, which contribute to a higher survival rate for these patients [52, 53]. Studies have shown that delays in starting treatment can increase the risk of mortality for cancer patients [54]. These results support the hypothesis that patients with higher hospital expenses may be more likely to start treatment more quickly or may have access to better treatment alternatives, significantly contributing to improved survival rates [52]. Additionally, our study revealed that patients treated in medium-complexity services had a higher risk of death compared to those treated in high-complexity services. This highlights the importance of early diagnosis and timely treatment, as hospitalization in medium-complexity services may be related to the care of patients with palliative or terminal treatment, where disease cure is not the main goal [55].

Effective strategies are needed to reduce mortality from CC. One recommended strategy is to enhance screening and ensure adequate coverage for early diagnosis, which can significantly reduce the risk of late diagnoses [13, 14], especially in age groups recommended by internationally recognized organizations, such as the World Health Organization [56]. This may include measures to facilitate access to early diagnosis and treatment. Another crucial strategy is to reduce the time between diagnosis and the start of treatment, as studies show that long intervals between these stages are associated with worse prognoses [40]. Implementing these strategies is essential and can have significant impacts on reducing CC mortality, as well as improving other critical factors such as treatment complexity, hospital costs, and the need for ICU admissions.

Despite the limitations related to the use of secondary data from the SUS SIH, the lack of detailed data on the clinical condition of patients during admission and hospitalization, and the limitation of sociodemographic variables, we were able to identify an overview of the survival of women undergoing treatment for CC in the state of Mato Grosso. In Brazil, there is a specific database for monitoring cancer patients during hospitalization, which is specific to Cancer Hospital Registries (RHC), an important tool in cancer surveillance, assisting in the oncology care network and cancer mortality, thus allowing understanding and identifying the situation of cancer morbidity and mortality [57]. However, despite the obligation to include data in the RHC being mandatory for all High Complexity Oncology Care Units (REF), in Brazil, there is a lag between hospitalization recorded in the SIH and the RHC. Regarding inference for the Brazilian population, it is necessary to consider regional characteristics and the organization of the local health system, as it is the responsibility of the municipal health manager to ensure cancer screening, early diagnosis, and referral for high-complexity treatment. More studies are needed that include a greater number of predictive data, thus increasing the explanation of the phenomenon studied. Although there are these limitations, the findings are fundamental for clinical decisions, personalized interventions, and improvements in healthcare. These insights can guide health policies and highlight the importance of specialized and high-complexity care to improve patient outcomes.

## Conclusion

This study reveals that patients hospitalized in the SUS with CC face significant survival challenges, emphasizing the importance of targeted interventions to improve their survival. Factors such as advanced age, lack of surgical procedures, hospital service complexity, ICU admission, and hospital costs were identified as critical predictors of mortality during hospitalization. These insights reinforce the need for continued investments in healthcare infrastructure and professional training to address the unique challenges faced by cancer patients in regions like Mato Grosso, especially considering its geographical diversity and predominance in agribusiness.

## Electronic supplementary material

Below is the link to the electronic supplementary material.

Supplementary Material 1

## Data Availability

In this study, publicly available datasets (SIH, Mato Grosso) were analyzed. These data are open access and freely available online at the following link: http://appweb3.saude.mt.gov.br/dw/pesquisa/tema.
