# Supplementary material for: Time to death from cervical cancer and its predictors in hospitalized patients: a survival approach study in Mato Grosso, Brazil
Source: World J Surg Oncol. 2024 Oct 9;22:269. doi: 10.1186/s12957-024-03518-y (PMC11463162; doi:10.1186/s12957-024-03518-y)
Supplement: Supplementary file 1 — Supplementary Material 1 [file 12957_2024_3518_MOESM1_ESM.docx]

**Suplements**

**S1.** Timetable of death for Cervical Cancer Patients

| **Time (in days)** | **n.risk** | **n.event** | **survival** | **std.err** | **95% CI** |
| --- | --- | --- | --- | --- | --- |
| 0 | 3493 | 19 | 0.995 | 0.00124 | 0.992, 0.997 |
| 1 | 3385 | 58 | 0.983 | 0.0022 | 0.979, 0.987 |
| 14 | 284 | 236 | 0.710 | 0.0177 | 0.676, 0.745 |
| 24 | 89 | 33 | 0.575 | 0.0265 | 0.526, 0.630 |
| 33 | 31 | 12 | 0.437 | 0.0421 | 0.362, 0.528 |
| 60 | 3 | 4 | 0.251 | 0.0824 | 0.132, 0.478 |
| 70 | 1 | 0 | 0.251 | 0.0824 | 0.132, 0.478 |

**S2.** Cumulative risk of death for Cervical Cancer Patients


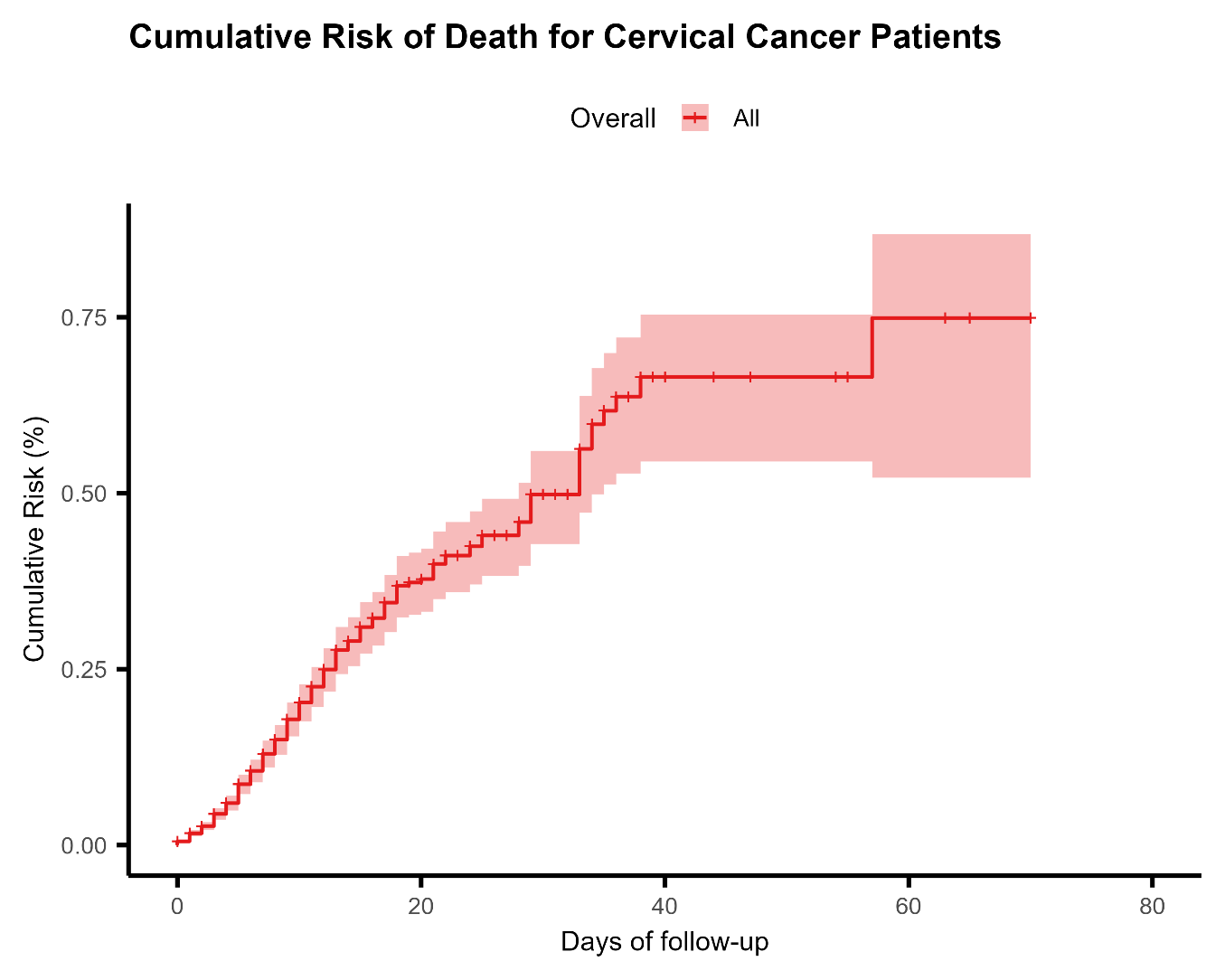


**Fig 3**. Cumulative risk of death for Cervical Cancer Patients

**S3.** Schoenfeld Residuals Analysis


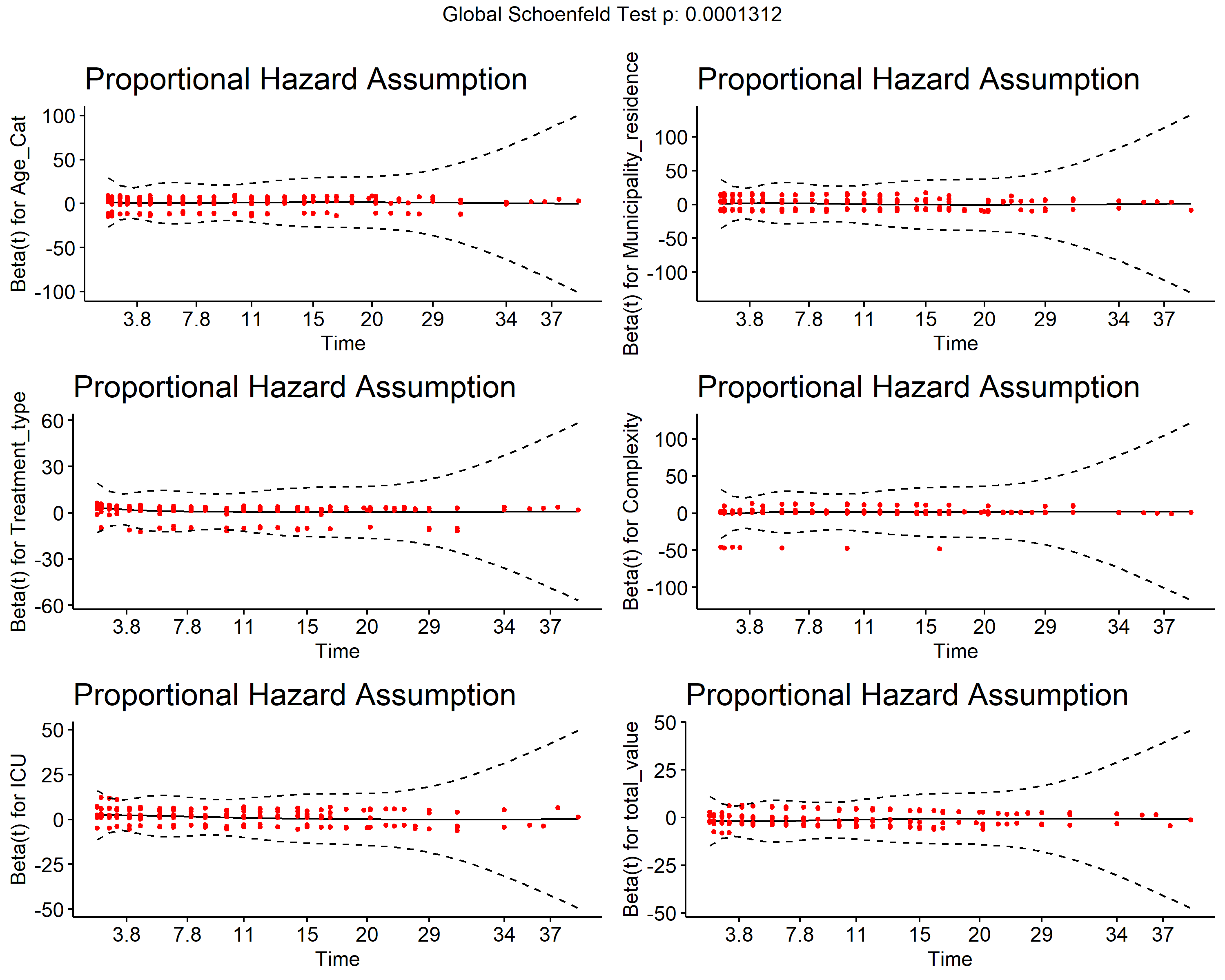


**Fig 4.** Schoenfeld Residuals Analysis
